# Supplementary material for: Transparent proton transport through a two-dimensional nanomesh material
Source: Nat Commun. 2019 Sep 3;10:3971. doi: 10.1038/s41467-019-11899-y (PMC6722077; doi:10.1038/s41467-019-11899-y)
Supplement: Supplementary file 1 — Supplementary Information [file 41467_2019_11899_MOESM1_ESM.pdf]

Supplementary Information

Transparent proton transport through two-dimensional nanomesh material

Jiyu Xu[1,2,5]†, Hongyu Jiang[1,2,5]†, Yutian Shen[1,2,5], Xin-Zheng Li[3,4], E.G. Wang[1,3,4,5*] & Sheng Meng[1,2,4,5*]

[1]Beijing National Laboratory for Condensed Matter Physics and Institute of Physics, Chinese Academy of Sciences, Beijing 100190, People's Republic of China

[2]School of Physical Sciences, University of Chinese Academy of Sciences, Beijing 100049, People's Republic of China

[3]State Key Laboratory for Mesoscopic Physics and School of Physics, Peking University, Beijing 100871, People's Republic of China

[4]Collaborative Innovation Center of Quantum Matter, Beijing 100871, People's Republic of China

[5]Songshan Lake Materials Laboratory, Dongguan, Guangdong 523808, People's Republic of China

†These authors contributed equally to this work. Correspondence and requests for materials should be addressed to E.G.W. (egwang@iphy.ac.cn) and S.M. (smeng@iphy.ac.cn)

Table of contents

1. **Supplementary Figures 1–12……………………………………………………S3**

2. **Supplementary Tables 1–2………………………………………………………S9**

3. **Supplementary Notes 1-6 ……………………………………………..……S11**

4. **Supplementary Method 1…………………………………………………...S15**

5. **Supplementary References……………………………………….……………S16**

Supplementary Figures

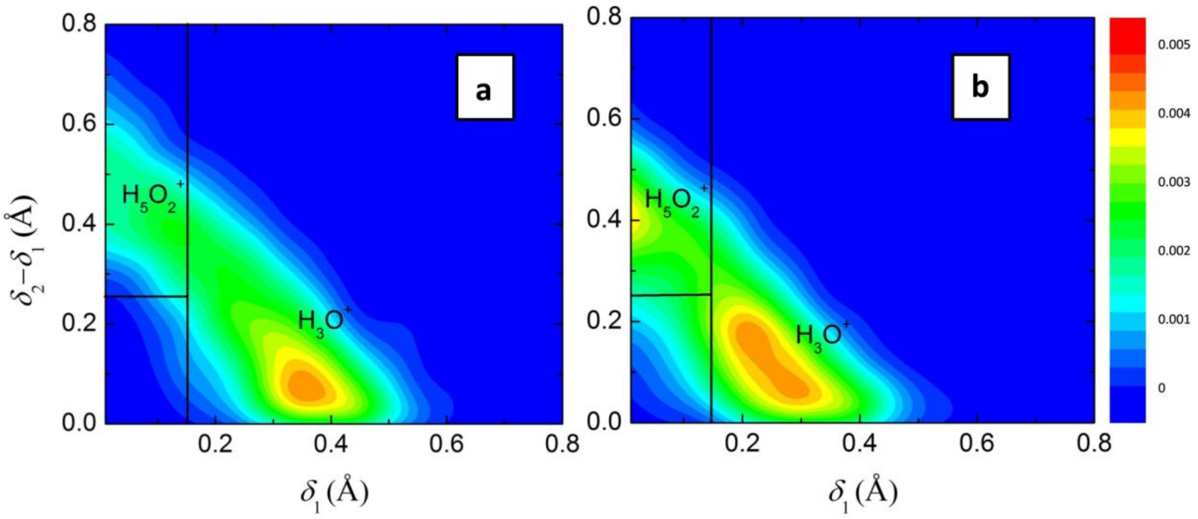

Supplementary Figure 1. Definition of proton complexes. The probability distribution of proton complex as a function of transfer coordinate δ_1 and δ_2 sampled in AIMD (a) and PIMD (b) simulations under electric field.

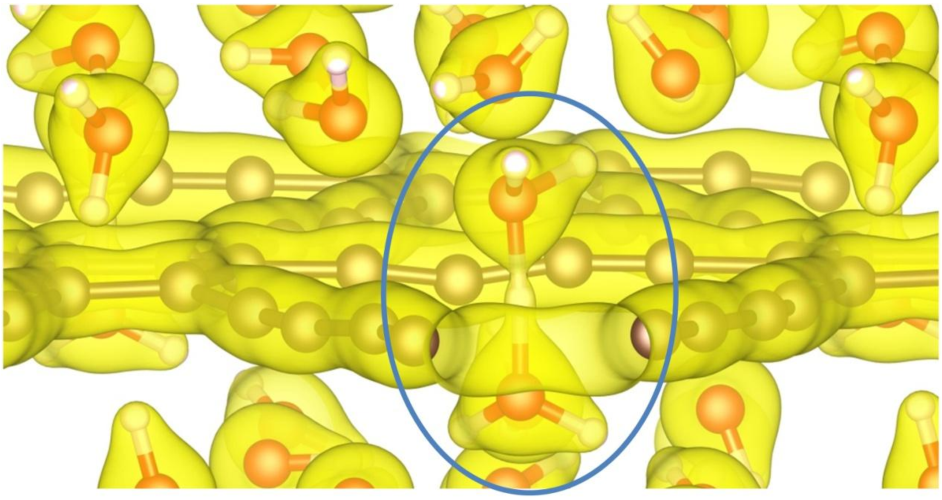

Supplementary Figure 2. Charge density distribution of a trans-membrane $H_5O_2^+$ complex.

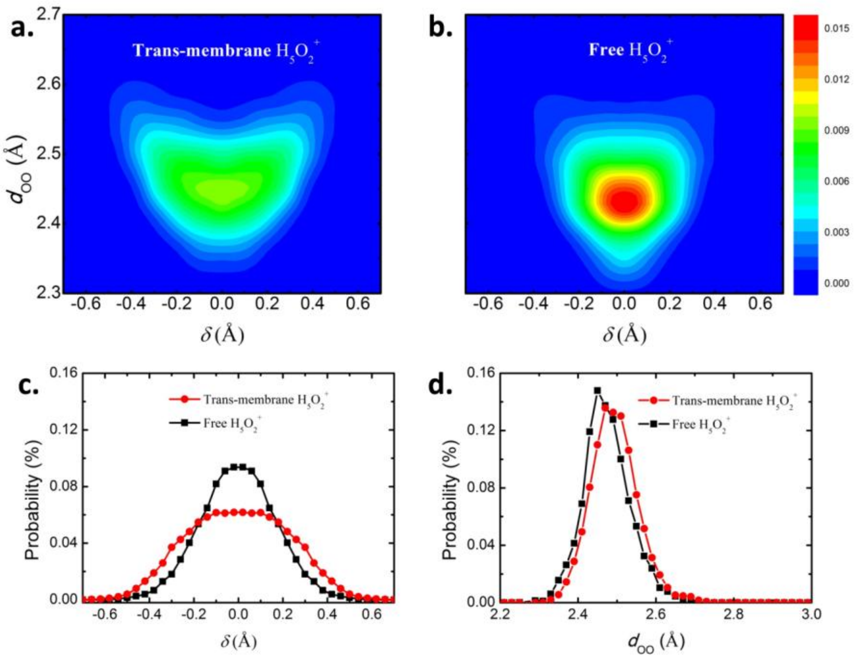

Supplementary Figure 3. Probability distribution of free $H_5O_2^+$ complex and trans-membrane $H_5O_2^+$ complex in vacuum. Probability distribution of the excess proton as a function of δ and d_{OO} for trans-membrane $H_5O_2^+$ (a) and free $H_5O_2^+$ (b) in vacuum. The distribution of (c) δ and (d) d_{oo} for excess proton in trans-membrane $H_5O_2^+$ and free $H_5O_2^+$ respectively.

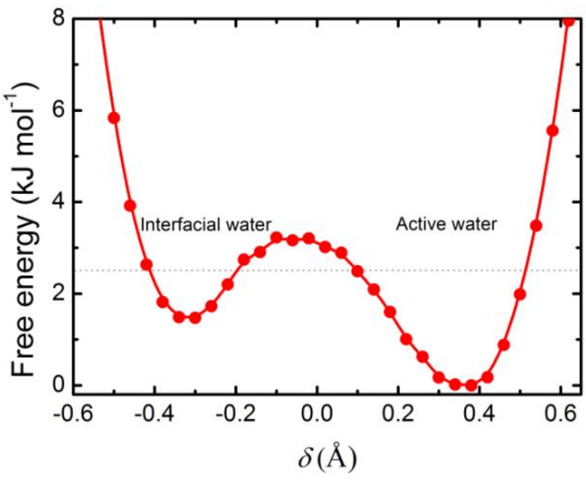

Supplementary Figure 4. Free energy profile for the excess proton transfer between the active water molecule and the interfacial water molecule. The k_BT is marked with a horizontal dashed line.

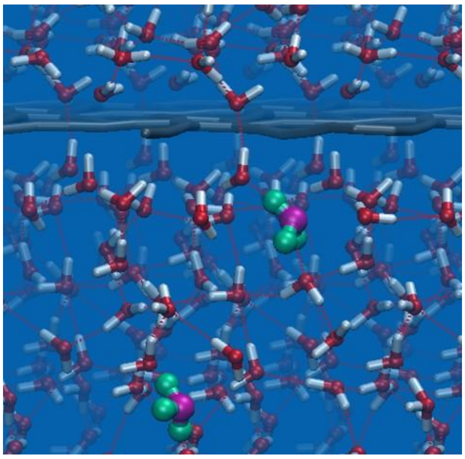

Supplementary Figure 5. A snapshot in AIMD simulations under electric field.

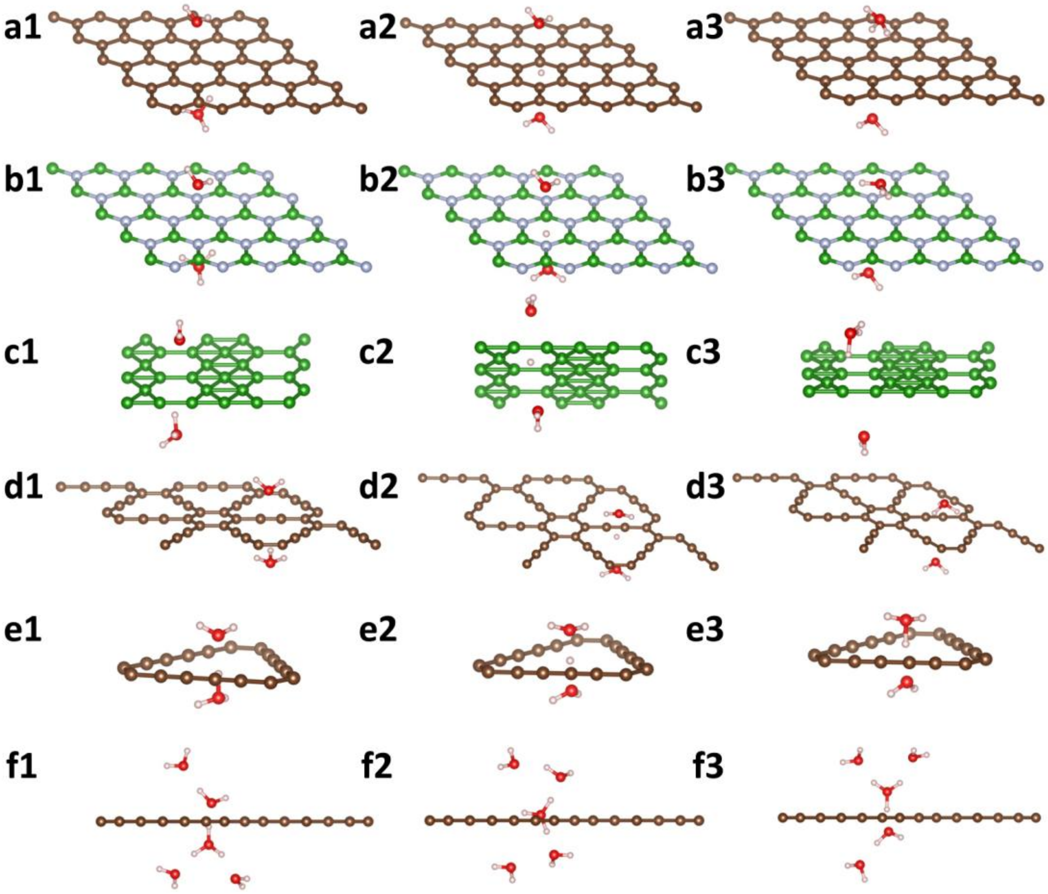

Supplementary Figure 6. CI-NEB calculations of proton transport across various 2D materials. The initial, intermediate and final configurations in CI-NEB calculations of proton transfer for graphene (a), h-BN (b), β-boron (c), graphyne-1 (d) and graphdiyne (e), respectively. The initial, intermediate and final configurations in CI-NEB calculations of H_3O^+ transport for graphdiyne (f). Color code: C, gray; H, white; O, red; B, green; N, silver.

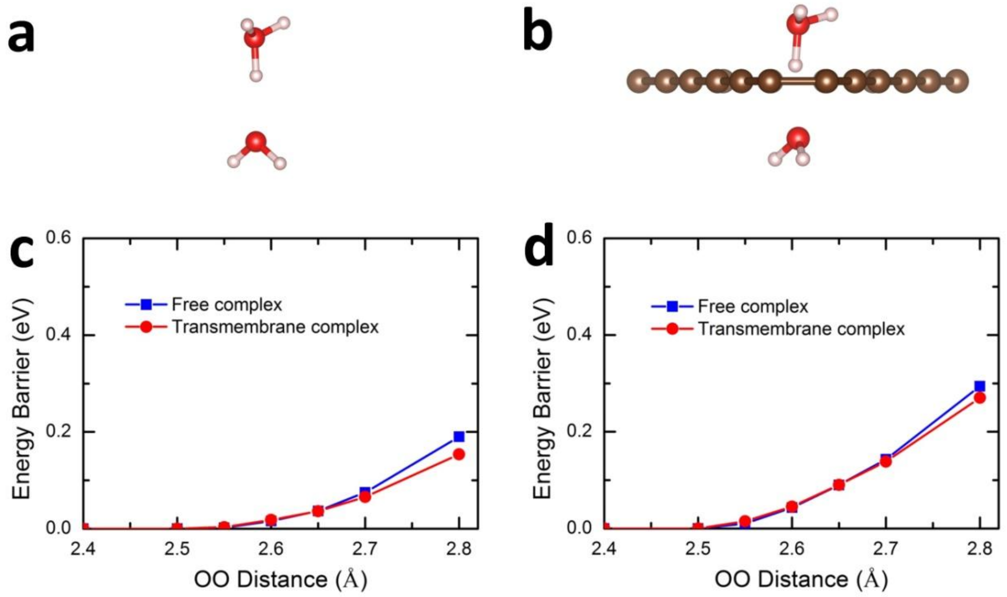

Supplementary Figure 7. Proton transparency of graphdiyne membrane. Structural models of (a) free H_3O^+-H_2O complex and (b) trans-membrane H_3O^+-H_2O complex. Energy barriers of proton transfer in free and trans-membrane H_3O^+-H_2O complex calculated with (c) BLYP-D3 and (d) B3LYP-D3.

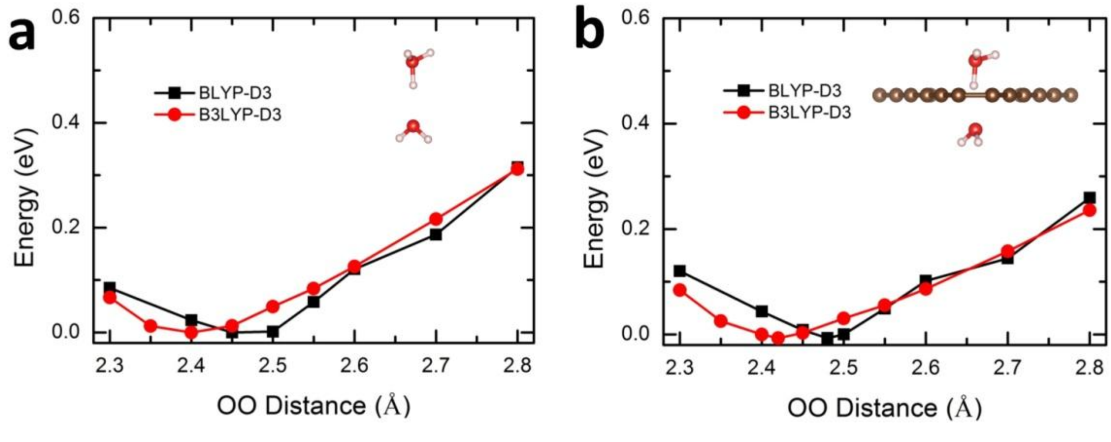

Supplementary Figure 8. Energy profile as the function of OO distance in (a) free and (b) trans-membrane proton complex.

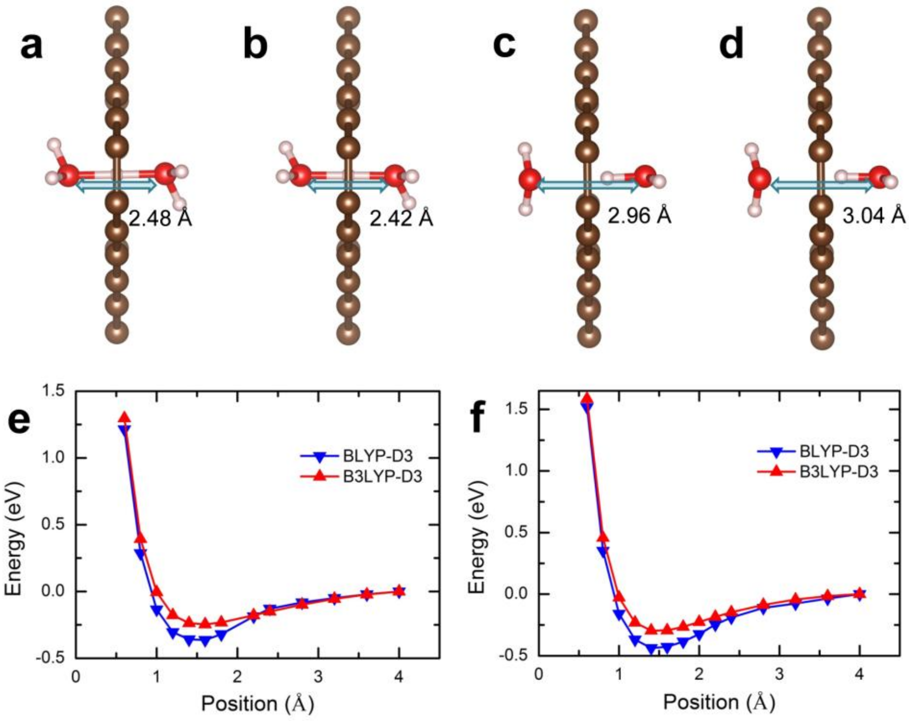

Supplementary Figure 9. Structures and energetics of trans-membrane proton complex and $(H_2O)_2$. Optimized trans-membrane proton complexes calculated by (a) BLYP-D3 and (b) B3LYP-D3 functional. Optimized trans-membrane $(H_2O)_2$ structures calculated by (c) BLYP-D3 and (d) B3LYP-D3 functional. Both functionals give similar water structures. Energy profiles of the right (e) and left (f) water molecule in trans-membrane $(H_2O)_2$ structure in Supplementary Figure 9d.

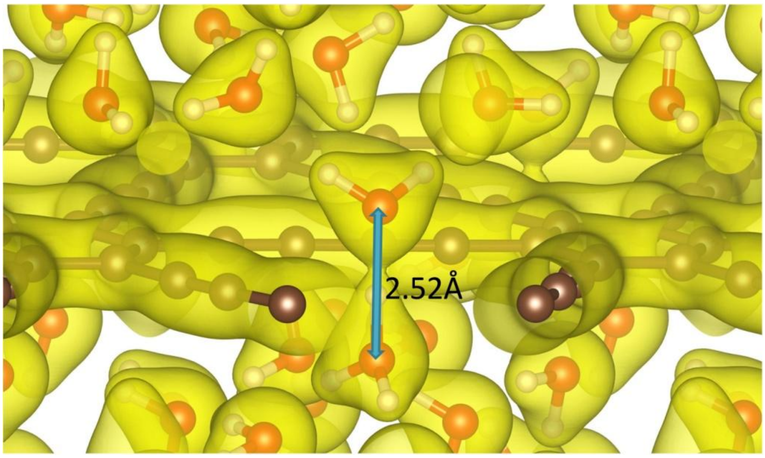

Supplementary Figure 10. Optimized structure with B3LYP-D3 and the charge density distribution of graphdiyne immersed in water.

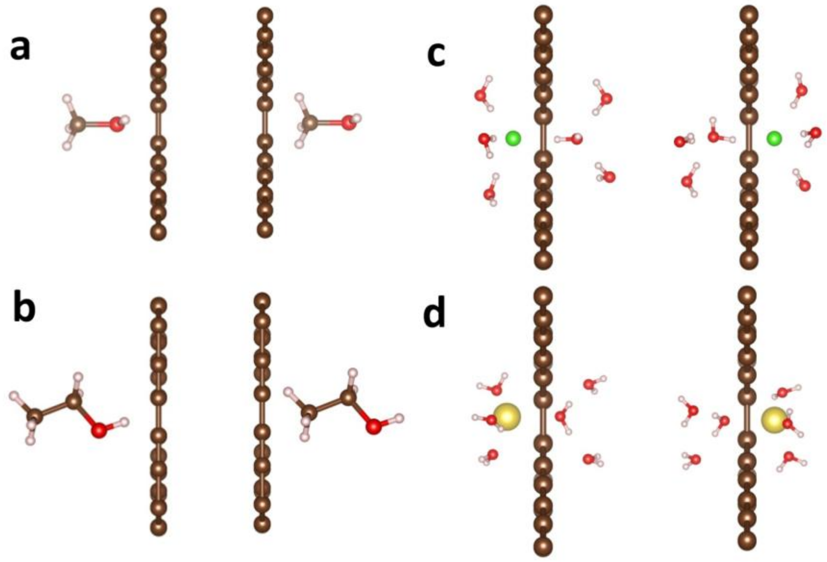

Supplementary Figure 11. Scanning-path calculations of solutes across graphdiyne membrane. Configurations in scanning-path calculations for CH_3OH (a), CH_3CH_2OH (b), Cl^- (c) and Na^+ (d) passing through nanopore on graphdiyne membrane. Color code: C, gray; H, white; O, red; Cl^-, green; Na^+, yellow.

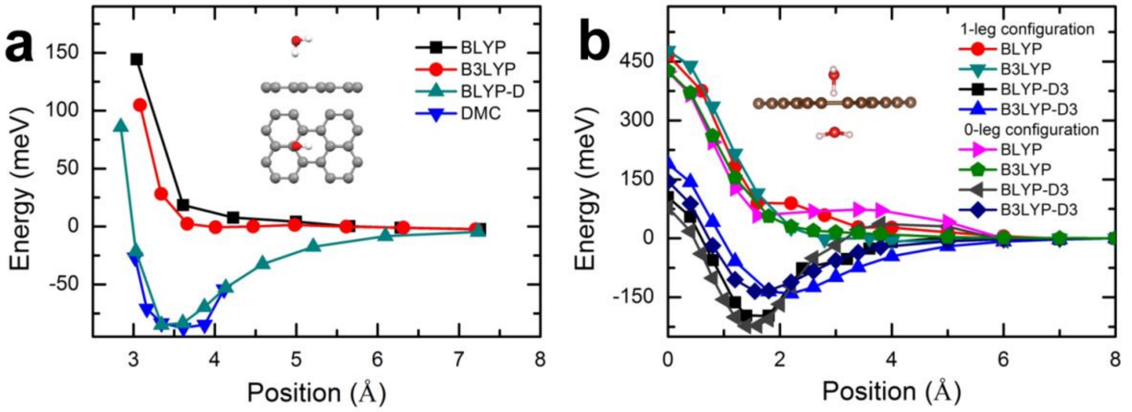

Supplementary Figure 12. The necessity of dispersion-correction in simulating water-carbon interactions. Energy profiles of water adsorption on graphene with one OH bond pointing to the surface (a). The inset exhibits the adsorption structures. Energy profiles of water adsorption on graphdiyne (b). The inset exhibits the adsorption structures, and the water molecules above and below graphdiyne membrane are defined as 1-leg and 0-leg molecules, respectively. The BLYP, B3LYP and BLYP-D results of water adsorption on graphene are taken from Ref. 1, and DMC results from Ref. 2, while all other data are calculated in the present work.

Supplementary Tables

**Supplementary Table 1 Adsorption distances and adsorption energies for 0-leg configuration of water molecule adsorption on graphene.**		
Method	Adsorption distance (Å)	Adsorption energy (meV)
DMC[2]	3.10	90
p-CCSD(T) [2]		84
RPA+GWSE[2]	3.05	90
CCSD(T)[3]	3.06	108
BLYP-D3[4]	~3.08	~88
B97M-rV[4]	~2.94	~127
DFT/CC[5]	3.01	90

**Supplementary Table 2 Adsorption distances and adsorption energies for 1-leg configuration of water molecule adsorption on graphene.**		
Method	Adsorption distance (Å)	Adsorption energy (meV)
DMC[2]	3.46	92
p-CCSD(T) [2]		76
RPA+GWSE[2]	3.45	87
DMC[1]	3.4 - 4.0	70
BLYP-D[1]	3.47	87
BLYP-D3[4]	~3.29	~137
B97M-rV[4]	~3.34	~127
DFT/CC[5]	3.35	125
vdw-DF2[6]	3.42	123
vdw-DF2^{c09x} [6]	3.42	78
optB86b-vdw[6]	3.35	143

Supplementary Note 1

Definition of proton complex based on transfer coordinate δ of protons

We define transfer coordinate δ of each proton as the distance difference between the proton and its two nearest oxygen atoms (O1 and O2), $\delta = d_{HO1} - d_{HO2}$. The excess proton is the proton with the smallest transfer coordinate δ. Then, we define the species of proton complex according to the transfer coordinate δ of protons. Once we find the excess proton according to the smallest δ, we find the two nearest oxygen atoms. Then, we calculate the transfer coordinates of the bonded four other hydrogen atoms. The proton with the smallest δ in the four hydrogen atoms is defined as the second excess proton. We define a H_3O^+ complex if the transfer coordinate δ_1 of excess proton is greater than 0.15 Å. We define a $H_5O_2^+$ complex if the transfer coordinate δ_1 of excess proton is less than 0.15 Å, and simultaneously the transfer coordinate δ_2 of the second excess proton is 0.25 Å greater than transfer coordinate δ_1 of excess proton. The other configurations correspond to complicated proton complex, *e.g.* $H_7O_3^+$ complex. Then, we define the position of proton complex according to the geometrical center.

Supplementary Note 2

Simulation of proton in bulk water

For simulations of proton in bulk water, 33 water molecules with one H_3O^+ complex were employed in a 10 $Å^3$ periodic cubic box. A 10-ps simulation was performed to sample proton transfer in bulk water.

Supplementary Note 3

Ab initio molecular dynamics simulations of free $H_5O_2^+$ complex and trans-membrane $H_5O_2^+$ complex in vacuum

Two 20-ps-long AIMD simulations were performed to test the stability and to sample the configurations of free $H_5O_2^+$ complex and trans-membrane $H_5O_2^+$ complex in vacuum. The two structures are both stable during the simulations. The distribution of inter proton (also the excess proton here) in trans-membrane $H_5O_2^+$ exhibits a broad

and planar distribution compared to that in free $H_5O_2^+$ complex, which attributes to the steric effect of membrane. Therefore, the trans-membrane $H_5O_2^+$ complex is a stretched but still stable $H_5O_2^+$ complex essentially.

Supplementary Note 4

Benchmark calculations with dispersion-corrected hybrid functional B3LYP-D3

We calculated the energy barrier of proton transfer in free and trans-membrane H_3O^+-H_2O complex with climbing image nudged elastic band method with BLYP-D3 in CP2K. Then, we performed benchmark calculations with dispersion-corrected hybrid functional B3LYP-D3 in VASP.[7] The structures from BLYP-D3 are used for energy calculations in B3LYP-D3. The two oxygen atoms are equidistant from the graphdiyne membrane.

Supplementary Figure 7a-b show the models used here. Supplementary Figure 7c-d show the energy barriers of two proton transfer processes calculated with two functionals. The energy barrier of zero means the formation of $H_5O_2^+$ complex. Despite the numerical differences in the two functionals, both functionals give the nearly same energy barrier profiles of proton transfer in free and trans-membrane H_3O^+-H_2O complex. Both demonstrate the negligible effect of graphdiyne membrane on proton transfer and demonstrate the proton transparency property of graphdiyne membrane. Despite that a little larger energy barrier is obtained in B3LYP-D3, we note that the proton transparency property is based on the comparison between the energy barrier of proton transfer in the two systems (with and without graphdiyne), not on the comparison between different functionals. Besides, the barrier of 0.27 eV is still much smaller than these of other 2D membranes.

Extra optimization calculations were performed for the free and trans-membrane complexes above with B3LYP-D3. Then, the results of stable proton complex with OO distance are compared with those from BLYP-D3. Below 2.55 Å, all four complexes exist as $H_5O_2^+$ complexes. Thus, both functionals give rise to the consistent proton species with OO distance for the free and trans-membrane complexes.

Supplementary Figure 8 exhibits the energy profiles of stable proton complex with OO distance. Consistent energetics are obtained with both functionals. Especially, both functionals demonstrate the stability of trans-membrane $H_5O_2^+$ complex in Supplementary Figure 8b, and the most stable trans-membrane complexes are shown in Supplementary Figure 9a-b. The negligible effect of graphdiyne membrane is demonstrated here. Furthermore, we can see a decrease of minimum position by 0.05-0.1 Å in B3LYP-D3 for both complexes. We note that the decrease of OO distance leads to the smaller energy barrier of proton transfer in NEB calculations. Thus, the decrease of minimum position compensates the little larger energy barrier of proton transfer in B3LYP-D3.

At the same time, we optimized the neutral trans-membrane structure of two water molecules. Supplementary Figure 9c-d show the nearly same optimized structures. We performed additional calculations of the two water molecule structures in Supplementary Figure 9d. We fixed the left water molecule in Supplementary Figure 9d and calculated the energy profiles of the right water molecule with both functionals, then calculated the energy profiles of the left water molecule in the same way. As shown in Supplementary Figure 9e-f, nearly the same minimum positions are obtained with both functionals for the left and right molecule respectively. Despite BLYP-D3 overestimates the interaction energy by ~0.14 eV, we note that the minimum position, which corresponds to the OO distance and reflects the characteristic interfacial water structure dominating trans-membrane proton transport, remains the same for both functionals. Thus, BLYP-D3 describes well the interfacial water structure and the proton energetics, attaining the accuracy of B3LYP-D3.

To include the aqueous effects in benchmark calculations, we extract from the trajectories in *ab initio* molecular dynamics simulations a snapshot in which proton diffuses close to graphdiyne nanopore, and perform geometry optimization calculation with hybrid functional B3LYP-D3. Supplementary Figure 10 shows the optimized structure and charge density distribution of the system. The OO distance is 2.52 Å and the angle H-O…O is 2°. A hydrogen bond is defined if the O-O distance is less than 3.5 Å and simultaneously the angle H-O...O is less than 30°. Thus, the trans-membrane hydrogen bond is explicitly formed across graphdiyne membrane. Thus the existence of the transport channel is confirmed with hybrid B3LYP-D3, which is the cornerstone

of proton transparency property. Meanwhile, excess proton does not bond to the pore rim even in hybrid functional calculations, which demonstrates the spatial constraint effect of nanopore for trans-membrane proton transfer.

The excess proton here is the one in trans-membrane hydrogen bond with δ of 0.39 Å and d_{OO} of 2.52 Å, which corresponds to the step i) of trans-membrane proton transfer. According to the classification standards in Supplementary Note 1 and Supplementary Figure 1, the excess proton here is H_3O^+ complex, which corresponds well to the features of H_3O^+ complex shown in Figure 4b calculated by BLYP-D3 functional (d_{OO}=2.52 Å and δ =0.36 Å).

Supplementary Note 5

Adsorption of water molecule on graphene

Supplementary Table 1 and Supplementary Table 2 exhibit the adsorption distances and adsorption energies of water molecules on graphene with 0-leg and 1-leg configurations respectively. The high-cost diffusion Monte Carlo, coupled cluster theory and random phase approximation give rise to various adsorption energies of a single water molecule absorbed on graphene for the two configurations. For the 0-leg configuration, BLYP-D3 shows the nice performances in describing this configuration. For the 1-leg configuration, overestimate of the adsorption energies is found for most DFT models, which is demonstrated in Ref. 4 and Ref. 6. Despite of the discrepancies of adsorption energies, the adsorption distances converge well for the both configurations.

Supplementary Note 6

The necessity of dispersion-correction in DFT calculations

Dispersion-correction is demonstrated to remedy the bad performances of DFT in describing the water-carbon interactions. Supplementary Figure 12a exhibits the energy profile of water monomer on graphene with BLYP-D,[1] consistent well with those with DMC.[2] Without dispersion-correction, repulsive interaction is obtained in

both GGA and hybrid functionals.[1] We performed benchmark calculations of water monomer adsorbed on the nanopore of graphdiyne membrane with and without D3. As shown in Supplementary Figure 12b, without D3 correction, repulsive interaction is also obtained with both GGA functional BLYP and hybrid functional B3LYP. Once D3 is included, the adsorption behavior is recovered with both functionals. Dispersion-corrected DFT gives reasonable description of interactions between water molecules and carbon materials.

Although the minimum positions are different by ~0.5 Å in 1-leg configurations for two dispersion-corrected functionals, the minimum positions are nearly the same in 0-leg configurations. Besides, the minimum positions are also nearly the same for adsorption of water molecules on both sides of the membrane (Supplementary Figure 9e-f), which reflects the same interfacial water structure. Furthermore, dispersion-corrected GGA indeed gives rise to reasonable interfacial structures between water and graphene or carbon-nanotubes in aqueous solutions.[8-10] Given the performance remediation of BLYP-D3 in many-body systems, we think that the discrepancies of different functionals may decrease due to the aqueous effects at liquid-solid interfaces, at least in the trans-membrane adsorption system here. We infer that many-body effects play an important role in water-carbon interactions.

Supplementary Method 1

Classical molecular dynamics simulations method

The simulation system considers a cubic box with dimensions of 38.0 × 32.9 × 300.0 Å, with 2540 water molecules located on two sides of membrane to sample the density profile and interfacial structure of water.

Classical MD simulation were performed with Gromacs 4.5.5 package.[11] Water molecule is modeled with SPC/E water model.[12] Non-bond interactions are modeled by Lennard-Jones (LJ) potentials with the water-substrate interaction parameters $\sigma_{C\text{-}O}$ = 3.02 Å, $\varepsilon_{C\text{-}O}$ = 1.734 kJ mol^{-1}.[13] The long-rang electrostatic interaction are computed with particle mesh ewald summation method.[14] A 6-ns-long simulation were performed

at 300 K with NVT ensembles after optimization and a timestep of 1 fs. Berendsen thermostat[15] is chosen to control the temperature.

Supplementary References

1. Ma, J. et al. Adsorption and diffusion of water on graphene from first principles. *Phys. Rev. B* **84,** 033402 (2011).
2. Brandenburg, J.G. et al. Physisorption of water on graphene: subchemical accuracy from many-body electronic structure methods. *J. Phys. Chem. Lett.* **10,** 358 (2019).
3. Voloshina, E., Usvyat, D., Schütz, M., Dedkov, Y. & Paulus, B. On the physisorption of water on graphene: a CCSD(T) study. *Phys. Chem. Chem. Phys.* **13,** 12041–12047 (2011).
4. Ajala, A. O., Voora, V., Mardirossian, N., Furche, F. & Paesani, F. Assessment of density functional theory in predicting interaction energies between water and polycyclic aromatic hydrocarbons: from water on benzene to water on graphene. *J. Chem. Theory Comput.* **15,** 2359−2374 (2019).
5. Rubeš, M., Nachtigall, P., Vondrášek, J. & Bludsky, O. Structure and stability of the water-graphite complexes. *J. Phys. Chem. C* **113,** 8412–8419 (2009).
6. Hamada, I. Adsorption of water on graphene: A van der Waals density functional study. *Phys. Rev. B* **86,** 195436 (2012).
7. Kresse, G. & Furthmuller, J. Efficient iterative schemes for ab initio total-energy calculations using a plane-wave basis set. *Phys. Rev. B* **54,** 11169 (1996).
8. Ruiz-Barragan, S., Muñoz-Santiburcio, D. & Marx, D. Nanoconfined water within graphene slit pores adopts distinct confinement-dependent regimes. *J. Phys. Chem. Lett.* **10,** 329−334 (2019).
9. Li, H. & Zeng, X. C. Wetting and interfacial properties of water nanodroplets in contact with graphene and monolayer boron-nitride sheets. *ACS Nano* **6,** 2401 (2012).
10. Li, H., Francisco, J. S. & Zeng, X. C. Unraveling the mechanism of selective ion transport in hydrophobic subnanometer channels. *Proc. Natl. Acad. Sci. U. S. A.* **112,** 10851-10856 (2015).
11. Pronk, S. et al. GROMACS 4.5: A high-throughput and highly parallel open source molecular simulation toolkit. *Bioinformatics* **29,** 845-854 (2013).
12. Berendsen, H., Grigera, J. & Straatsma, T. The missing term in effective pair potentials. *J. Phys. Chem.* **91,** 6269-6271 (1987).
13. Xu, J. et al. Water transport through subnanopores in the ultimate size limit: Mechanism from molecular dynamics. *Nano Res.* **12,** 587–592, (2019).
14. Darden, T., York, D. & Pedersen, L. Particle mesh Ewald: an N· log (N) method for Ewald sums in large systems. *J. Chem. Phys.* **98,** 10089-10092 (1993).
15. Berendsen, H. J., Postma, J. P., van Gunsteren, W. F., DiNola, A. & Haak, J. Molecular dynamics with coupling to an external bath. *J. Chem. Phys.* **81,** 3684-3690 (1984).
